# Impact of plant domestication on rhizosphere microbiome assembly and functions

**DOI:** 10.1007/s11103-015-0337-7

**Published:** 2015-06-18

**Authors:** Juan E. Pérez-Jaramillo, Rodrigo Mendes, Jos M. Raaijmakers

**Affiliations:** Department of Microbial Ecology, Netherlands Institute of Ecology (NIOO-KNAW), P.O. Box 50, 6708 PB Wageningen, The Netherlands; Sylvius Laboratories, Institute of Biology, Leiden University, Sylviusweg 72, 2333 BE Leiden, The Netherlands; Laboratory of Environmental Microbiology, Brazilian Agricultural Research Corporation, Embrapa Environment, Rodovia SP 340 - km 127.5, Jaguariúna, 13820-000 Brazil

**Keywords:** Rhizosphere microbiome, Plant domestication, Wild relatives, Plant–microbe interactions

## Abstract

The rhizosphere microbiome is pivotal for plant health and growth, providing defence against pests and diseases, facilitating nutrient acquisition and helping plants to withstand abiotic stresses. Plants can actively recruit members of the soil microbial community for positive feedbacks, but the underlying mechanisms and plant traits that drive microbiome assembly and functions are largely unknown. Domestication of plant species has substantially contributed to human civilization, but also caused a strong decrease in the genetic diversity of modern crop cultivars that may have affected the ability of plants to establish beneficial associations with rhizosphere microbes. Here, we review how plants shape the rhizosphere microbiome and how domestication may have impacted rhizosphere microbiome assembly and functions via habitat expansion and via changes in crop management practices, root exudation, root architecture, and plant litter quality. We also propose a “back to the roots” framework that comprises the exploration of the microbiome of indigenous plants and their native habitats for the identification of plant and microbial traits with the ultimate goal to reinstate beneficial associations that may have been undermined during plant domestication.

## Introduction

Plants rely on their rhizosphere microbiome for functions and traits related to plant growth, development and health (Berendsen et al. 2012; Mendes et al. 2013). Members of the rhizosphere microbiome harbour a range of beneficial properties contributing to nutrient acquisition, enhanced stress tolerance, protection against soil borne pathogens and host immune regulation (Berendsen et al. 2012; Bakker et al. 2013; Mendes et al. 2013; Turner et al. 2013a; Berg et al. 2014; Lakshmanan et al. 2014). In this context, Cook et al. (1995) postulated that natural selection resulted in only few examples of plant genetic resistance against belowground pathogens and that plant rely, in part, on the natural defence provided by rhizosphere microorganisms. This is the case for natural disease suppressive soils where specific microbial consortia protect the host from infection (Mendes et al. 2011). Assuming that plants depend, at least in part, on the rhizosphere microbiome as a product of natural selection, modern cultivars of crop plants may have lost some of the traits needed to recruit host-specific root microbiota as compared to their wild relatives, which are genetically more diverse and adapted to pre-agricultural soils (Wissuwa et al. 2009; Bulgarelli et al. 2013). Whether the ability of crop plants to recruit beneficial rhizosphere microbes is undermined by plant domestication and plant breeding is not well known to date. In this review, we discuss the potential influence of plant domestication on rhizosphere microbiome assembly and function, focusing on how domestication may have impacted the ability of modern crops to establish beneficial interactions with the rhizosphere microbiome. Finally, we propose a framework for identification and recovery of beneficial plant–microbe interactions to meet the need for a more sustainable and productive agriculture.

## Plant domestication: changes and trade-offs

One of the biggest accomplishments in human history has been the domestication of plants, providing a more continuous food supply and promoting the conformation of sedentary agricultural groups (Purugganan and Fuller 2009). The process of plant domestication involves selection, modification and adoption of wild plants species with useful characteristics for human use (Gepts 2004). The first changes commonly associated with plant domestication were a large seed size, loss of seed dispersal mechanisms, and determinate growth and apical dominance (Gross and Olsen 2010). Other changes comprise the loss of seed dormancy, decrease of bitter substances in edible structures and changes in photoperiod sensitivity (Purugganan and Fuller 2009). Domestication also led to a reduction in genetic diversity of plant cultivars as shown for common bean (Bitocchi et al. 2012, 2013), rice (Ram et al. 2007) and wheat (Haudry et al. 2007). Genes associated with desirable phenotypes underwent a diversity loss because only the desired alleles were spread in the subsequent progenies, whilst unwanted diversity of the same allele was inadvertently suppressed (Doebley et al. 2006). In addition, genomic regions next to the target genes suffered selective sweeps as was shown for the adjacent regions of the *Y1* phytoene synthase gene for endosperm colour in maize (Palaisa et al. 2004) and of the *Waxy* granule-bound starch synthase gene for amylose synthesis in rice (Olsen et al. 2006). Thus, a possible side effect of plant domestication is the loss of traits neglected during human selection. In a recent review, Chen et al. (2015) indicated that the ability of plants to deal with herbivorous insects is undermined in domesticated crops, in part as a consequence of changes in morphological traits and in levels of secondary metabolites, which make domesticated plants a better resource for insects as compared to wild relatives. Chen et al. (2015) further highlighted that domestication led to lower levels of volatile emissions as compared to wild relatives, which in turn may affect the attraction of natural enemies. Whether plant traits needed to recruit and sustain beneficial microbial populations in the rhizosphere was also negatively impacted remains to be elucidated.

**Fig. 1:**
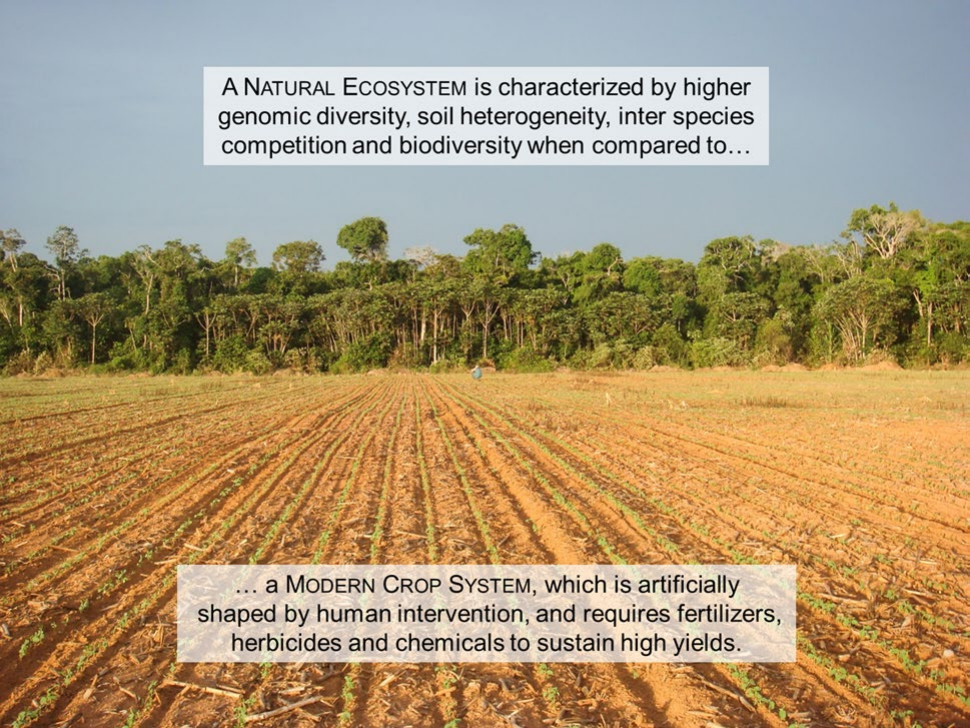
In this image, the natural ecosystem is illustrated by the native Amazon rainforest (*background*) that was converted to a modern crop system (*front*). This conversion leads to changes in the environment and use of management techniques ultimately impacting the rhizosphere microbiome assembly and functions (Photo by L. W. Mendes)

**Fig. 2:**
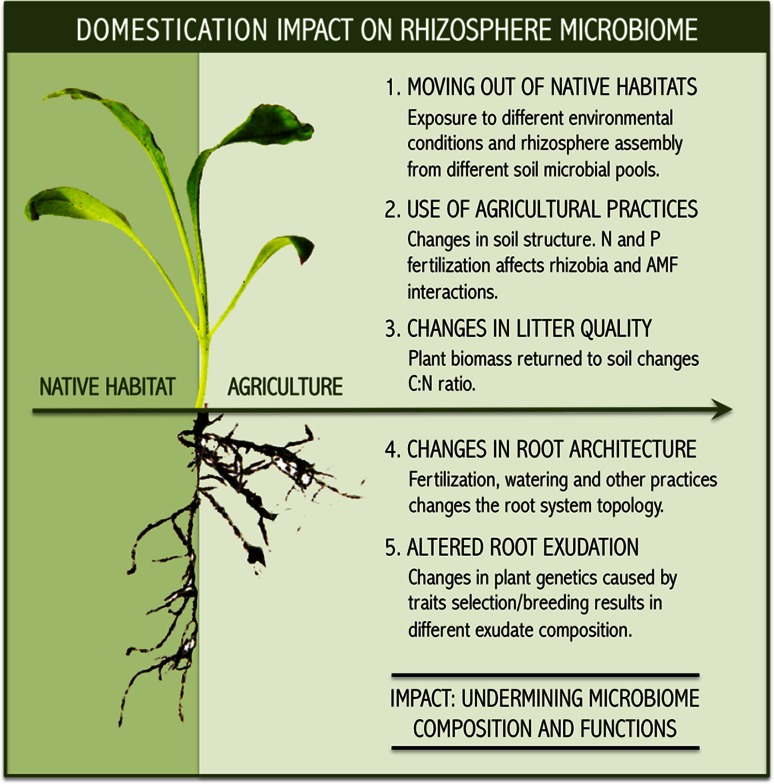
Changes associated to the domestication process affect plant traits and soil properties undermining rhizosphere microbiome composition and functions

Many of the changes in plant traits during domestication were accompanied by progressive changes in the environment and management practices (Fig. 1). Hence, plant domestication associated with anthropogenic interference to sustain high yields led to low self-support production systems with an increased need for external inputs such as chemical pesticides and fertilizers to overcome problems related to pests and diseases, vulnerability to abiotic stress and nutrient depletion (Matson et al. 1997). Moreover, the transition from natural to agricultural systems may have hampered beneficial interactions between plants and microbes due to loss of soil microbial diversity. For instance, it was shown that long-term nitrogen fertilization resulted in the evolution of less-mutualistic rhizobia, providing fewer benefits to the host (Weese et al. 2015). Nitrogen amendments have also been shown to suppress soil respiration and microbial biomass, promoting copiotrophs such as Actinobacteria and Firmicutes while reducing the abundance of oligotrophs such as Acidobacteria and Verrucomicrobia (Ramirez et al. 2012). This was substantiated by Rodrigues et al. (2013) who showed that conversion of the Amazon rainforest to agriculture resulted in biotic homogenization of soil bacterial communities and reduction of microbial diversity. Fierer et al. (2013) further showed that in a native tallgrass prairie ecosystem, bacterial communities did not resemble those harboured by the surrounding cultivated soils where Verrucomicrobia represented more than 50 % of the 16S rRNA sequences identified. Also soil attributes can be affected by plant domestication, which in turn influence the soil microbial community composition. García-Palacios et al. (2013) demonstrated, in microbial-rich and microbial-poor soils, that plant domestication increased litter quality, resulting in lower C:N ratio and higher NO_3_ availability. In addition to changes in the production systems, domesticated lineages experienced range expansions far beyond their centres of origin due to human migrations and trade (Purugganan and Fuller 2009). Hence, the lack of a co-evolutionary trajectory between plants, microbial communities and pathogens in dissimilar agricultural landscapes, made human interventions even more critical to maintain a healthy and productive crop (Fig. 2).

## Effect of plant genotype on rhizosphere microbiome assembly

Plants can modulate their rhizosphere microbiome in a host-dependent way. Each plant species promotes a particular set of rhizosphere microbes (Haichar et al. 2008; Turner et al. 2013b; Ofek et al. 2014). With an increase in the phylogenetic distance between plant species also differences in the composition of their rhizosphere microbial assemblages appear to increase (Wieland et al. 2001; Pongsilp et al. 2012; Bouffaud et al. 2014). Not only different plant species, but also different genotypes of the same species may differ in their rhizosphere microbiome composition. For example, Weinert et al. (2011) showed for three different potato cultivars that a portion of the detected OTUs was cultivar-specific and that the Streptomycetaceae responded in a cultivar-dependent manner. Similar cultivar-dependent effects were observed for the rhizobacterial communities in the rhizosphere of young potato plants (Inceoglu et al. 2011). In a recent study with 27 modern maize inbred lines, grown in five field environments, Peiffer et al. (2013) showed that OTU richness was affected by maize genotypes and that the variation in β-diversity was partially explained by the maize genotype. Similarly, in a study with different barley genotypes, Bulgarelli et al. (2015) found that the host genotype accounts for approximately 5.7 % of the variance in the rhizosphere microbiome composition. In sweet potato (*Ipomoea batatas*), *Sphingobium*, *Pseudomonas*, *Acinetobacter*, *Stenotrophomonas*, and *Chryseobacterium* were enriched on the low starch genotype as compared to two high starch genotypes (Marques et al. 2014). Next to genotype-specific effects, also the plant developmental stage is a strong driver shaping the rhizobacterial community structure. In soybean, *Bradyrhizobium*, *Bacillus* and *Stenotrophomonas* were more abundant at the flowering stage as compared to vegetative and mature stages (Sugiyama et al. 2014a). For fungal communities, however, no significant effects of the soybean growth stage were detected (Sugiyama et al. 2014b). These effects, however, are not general as some studies highlighted a stronger selective rhizosphere effect at young plant growth stages (Gomes et al. 2001; Jin et al. 2009; Micallef et al. 2009a; Xu et al. 2009; Chaparro et al. 2014) whilst other studies documented stronger selective effects on the microbiome at flowering (Smalla et al. 2001; Inceoglu et al. 2010).

## Plant root exudates and the recruitment of beneficial microbes

Plants actively release exudates, volatile compounds, border cells and polymers into the soil, a process referred to as rhizodeposition (Jones et al. 2009). Root exudates are composed of low-molecular weight compounds, i.e. sugars, amino acids, organic acids, phenolics, secondary metabolites, and high-molecular weight compounds like proteins and mucilage (Badri and Vivanco 2009). For more details on the chemical diversity of compounds in the rhizosphere we refer to other reviews (Bais et al. 2006; Badri et al. 2009; Moe 2013; Weston and Mathesius 2013; Baetz and Martinoia 2014; Haichar et al. 2014).

Root exudates may impact the functioning of the microbial community. For instance, in soil amended with maize mucilage a higher production of N_2_O was recorded than in non-amended soil (Mounier et al. 2004). Likewise, additions of artificial root exudates (ARE) to a soil microcosm, mimicking maize exudates, promoted nitrate reduction and denitrification (Henry et al. 2008). A study with eight *Arabidopsis thaliana* accessions revealed that each accession released a particular set of exudate compounds and that each accession had a distinct rhizobacterial community composition based on RISA and 16S-TRFLP analyses (Micallef et al. 2009b). Some root exudates may impact the microbial community structure to a greater extent than other compounds as was shown for organic acids with a 10–22 fold increase in the bacterial taxa while sugars showed only a 2.5 fold increase (Shi et al. 2011). When Arabidopsis exudates collected from 18 to 21 days old plants were applied to a fallow soil, phenolic compounds had a significant positive correlation with the highest number of bacterial OTUs (742) whereas lower number of OTUs were found for amino acids (319), sugar alcohols (166), and sugars (161) (Badri et al. 2013). Root exudates such as flavonoids or strigolactones play key roles in symbiotic relationships between plants and rhizobia, mycorrhiza and also parasites (Jones et al. 2004; Bouwmeester et al. 2007; Bednarek et al. 2010; Wang et al. 2012; Haichar et al. 2014). Likewise, root exudates may impact specific groups of beneficial bacteria referred as plant growth promoting rhizobacteria (PGPR). For example, *Pseudomonas fluorescens* WCS365, a rhizosphere competent colonizer of tomato roots, was chemotactically attracted towards malic acid and citric acid exuded by tomato roots (de Weert et al. 2002). Also beneficial rhizobacterium *Bacillus subtilis* FB17 exhibited a positive chemotactic response towards L-malic acid. Interestingly, infection of Arabidopsis leaves with *Pseudomonas syringae* pv *tomato* induced an increased secretion of malic acid from the roots, promoting the colonization and biofilm formation by strain FB17 (Rudrappa et al. 2008). Furthermore, foliar pathogens or foliar treatment with microbe-associated molecular patterns (MAMPs) of Arabidopsis leaves promoted the expression of the root malic acid (MA) transporter (*ALMT1*), stimulating the colonization by *B. subtilis* strain FB17 (Lakshmanan et al. 2012). Malic acid and citric acid exuded by watermelon roots were shown to induce motility and root colonization by the PGPR *Paenibacillus polymyxa* SQR-21 (Ling et al. 2011). Similar effects of malic acid and citric acid were found for chemotaxis and biofilm formation by *Bacillus amyloliquefaciens* SQR9 in cucumber and for fumaric acid in promoting colonization of banana roots by *B. subtilis* N11 (Zhang et al. 2014). Also other compounds found in root exudates may recruit beneficial bacteria. The aromatic compound 2,4-dihydroxy-7-methoxy-2H-1,4-benzoxazin-3(4H)-one (DIMBOA) exuded by maize roots, showed a chemoattractant effect on and an increased root colonization by *Pseudomonas putida* KT2440 (Neal et al. 2012). Finally, plant derived compounds may also have an effect on the expression of bacterial antifungal biosynthetic genes. For instance, the expression of *phlA* and *pltA* genes in *P. fluorescens* CHA0, involved in the biosynthesis of the antifungal compounds 2,4-diacetylphloroglucinol (DAPG) and pyoluteorin (PLT) respectively, was induced or repressed by 40 different plant-derived compounds, including several plant phenolics and pectin (de Werra et al. 2011). Similarly, phenolic and organic acids exuded by barley plants infected with the fungus *Pythium ultimum* induced the expression of the *phlA* gene of *P. fluorescens* CHA0, presumably as a plant systemic response to deal with the pathogen (Jousset et al. 2011). Also *Zea mays* subsp. *parviglumis* and European maize lines emitted the volatile sesquiterpene (*E*)-β-caryophyllene via the roots attracting an entomopathogenic nematode in response to insect attack; North American lines failed to release this compound probably as a consequence of the breeding process (Rasmann et al. 2005; Köllner et al. 2008).

These results exemplify the potential of plants to recruit and activate, via specific components in root exudates, beneficial members of the rhizosphere microbiome. However, plant exudates may also exert a negative effect on belowground communities. In a study evaluating the effect of the invasive weed *Centaurea maculosa* on the composition of arbuscular mycorrhizal fungal (AMF) communities, the abundance and diversity of AMF was reduced compared to native grassland samples (Mummey and Rillig 2006). The same deleterious effect of *C. maculosa* was also shown for the overall soil fungal community. Broz et al. (2007) further observed that high density weed populations had a reduced fungal biomass and diversity as compared to low density weed populations mixed with native species. Badri and Vivanco (2009) suggested that root exudates released by invasive weeds disrupt the indigenous microbial communities probably through an antimicrobial effect. Although the available results are still limited, fragmentary and not conclusive, one may assume that plant domestication can lead to changes in root exudation profiles and thereby impact on the rhizosphere microbiome composition and function (Fig. 2).

## Effect of plant domestication on belowground interactions

### Undermined mycorrhizal symbiosis

The effect of domestication and plant breeding on belowground interactions with soil microorganisms was addressed by pioneering studies with wheat evaluating the ability of ancestors, landraces and modern genotypes to sustain mycorrhizal symbiosis (Kapulnik and Kushnir 1991). The mycorrhizal dependence (MD), i.e. the degree of dependence on mycorrhizal symbiosis for maximum plant growth and yield, was determined for wild and cultivated wheat genotypes. The results showed that a diploid wheat ancestor, *Triticum tauschii,* displayed a higher MD compared to tetraploid or modern hexaploid wheat genotypes (Kapulnik and Kushnir 1991). Hetrick et al. (1992) further showed that ancestors and primitive hexaploid wheat landraces benefitted more from mycorrhizal symbiosis than modern cultivars. Subsequently, Hetrick et al. (1993) determined that the ancestral genotype *T. tauschii* var. *strangulata,* the donor of the D genotype in hexaploid modern wheat, showed a higher MD as reported in previous studies, whilst AB genome ancestors did not show mycorrhizal dependence. In these studies, the highly fertile conditions used during the plant breeding process were proposed as a possible explanation for the reduced mycorrhizal dependence of modern genotypes. To support this observation, Hetrick et al. (1995) further showed that wheat varieties released before 1975 displayed a higher mycorrhizal responsiveness (MR), defined as the effect of the mycorrhizal symbiosis on plant growth as compared to plants without mycorrhiza, while those released after this date were less responsive. Accordingly, Zhu et al. (2001) also found a reduction in MR in Australian modern wheat cultivars as compared to old cultivars. However, these findings were recently contrasted in a meta-analysis of mycorrhizal responsiveness in wild and annual crop plants. Lehmann et al. (2012) found that newer genotypes were more mycorrhiza-responsive compared to the ancestral genotypes although less intensively colonized. A possible explanation for this observation is that ancient genotypes, and to a larger extent wild relatives, may have developed adaptations to low nutrient environments and are less dependent on mycorrhizal infection than newer genotypes (Koide et al. 1988). However, a decrease in the ability to sustain AMF symbiosis in modern cultivars has been also found for other crops. For instance, it was shown that domesticated breadfruit cultivars (*Artocarpus altilis*) were less able to support AMF as compared with wild ancestors as revealed by significant reductions of vesicular and arbuscular colonization (Xing et al. 2012). In maize, the response of four landraces and one hybrid to AMF in two different phosphorus (P) regimes was evaluated; two local landraces were significantly more colonized by AMF and acquired more phosphorus in shoots under low and medium P regimes as compared to the modern maize hybrid. Interestingly, one of the landraces presented an outstanding mycorrhizal colonization and presented the highest percentage increase in root volume under both P regimes (Sangabriel-Conde et al. 2014). The diversity of AMF in the roots of the four landraces and the hybrid was assessed through nested PCR of AMF rDNA and it was shown that the landrace with higher mycorrhizal colonization and P acquisition efficiency also presented the highest number of Glomeromycota OTUs (Sangabriel-Conde et al. 2015). The authors proposed that the adoption of native landraces of maize may preserve mycorrhizal symbiosis in these agricultural landscapes.

### Domestication effect on rhizobia and other microbes

The effect of plant domestication has also been assessed for the symbiosis between legumes and rhizobia. In a study with pea (*Pisum sativum)*, broad bean (*Vicia faba)* and several wild legumes from the genera *Vicia* and *Lathyrus*, grown in a non-agricultural soil, it was shown that the ability to interact with symbionts was limited for pea and broad bean as compared to promiscuous wild legumes that were able to exploit the diverse rhizobial community (Mutch and Young 2004). Similarly, it was found that *Cicer reticulatum*, ancestor of cultivated chickpea, showed association with a more diverse *Mesorhizobium* population than modern chickpea (Kim et al. 2014). In the legume–rhizobia symbiosis, Kiers and Denison (2008) described that plants can sanction less effective symbionts and invest more resources in highly efficient strains. In order to assess whether the ability to sanction non-effective rhizobia strains was also affected in the breeding process, six soybean cultivars representing 60 years of breeding were evaluated in the simultaneous presence of effective and ineffective rhizobia strains. Kiers et al. (2007) showed that newer cultivars had less seed yields as compared to older cultivars and also that the yield difference ratio, i.e. the ability of cultivars to reach their full symbiotic potential in the presence of mixed rhizobial strains, was higher for older cultivars as compared to newer cultivars.

For the effects of plant domestication on other rhizosphere microbes only few examples exist to date. Germida and Siciliano (2001) revealed that the rhizosphere bacterial community of ancient landraces was more diverse than that of two modern cultivars. In the rhizosphere of the ancestral landrace, Pseudomonads were the dominant genus and also higher numbers of *Aureobacter* were found as compared to modern cultivars (Germida and Siciliano 2001). Also in maize, the influence of its progenitor *Zea mays* subsp. *parviglumis* (Balsas teosinte) and two domesticated maize cultivars on the rhizosphere bacterial and fungal community composition was evaluated (Szoboszlay et al. 2015). Shannon’s and Simpson’s diversity indices for bacterial T-RLFP profiles were higher for teosinte compared with one domesticated cultivar and the same as the other cultivar and the control. Interestingly, the same domesticated cultivar with lower bacterial diversity also showed a lower fungal diversity compared with bulk soil controls (Szoboszlay et al. 2015).

## Domestication and changes in root architecture

Differences in root architecture between modern cultivars and their wild relatives have been described for a number of crops. For instance, cultivated lettuce (*Lactuca sativa*) produced a shallower root system compared with wild lettuce (*Lactuca serriola*). In cultivated lettuce an inadvertent selection of more laterals roots at the top of the tap root helps plants to respond to surface application of water and fertilizer in crop fields, whereas wild lettuce showed a root system able to access deeper portions of soil (Jackson 1995). Changes in root architecture have also been described for drought tolerant plant cultivars. For example, a drought tolerant accession of wild barley presented different root length, root dry weight and root volume compared with a modern cultivar, both under control and drought conditions (Naz et al. 2012, 2014). Similarly, the teosinte *Zea mays* subsp. *parviglumis* showed a higher root to shoot dry weight ratio and a higher number of very fine and thick roots than two domesticated maize cultivars. Although the Shannon’s and Simpson’s diversity indices for the bacterial communities were higher for teosinte compared with one the domesticated cultivar, the relative contribution of the root architecture for the observed microbiome differences was not investigated (Szoboszlay et al. 2015). It has been postulated that changes in root architecture due to breeding process may have an effect on the rhizosphere microbiome (Micallef et al. 2009b), however, more detailed studies will be needed to investigate this.

## Reinstating beneficial partnerships in modern crop cultivars

**Fig. 3:**
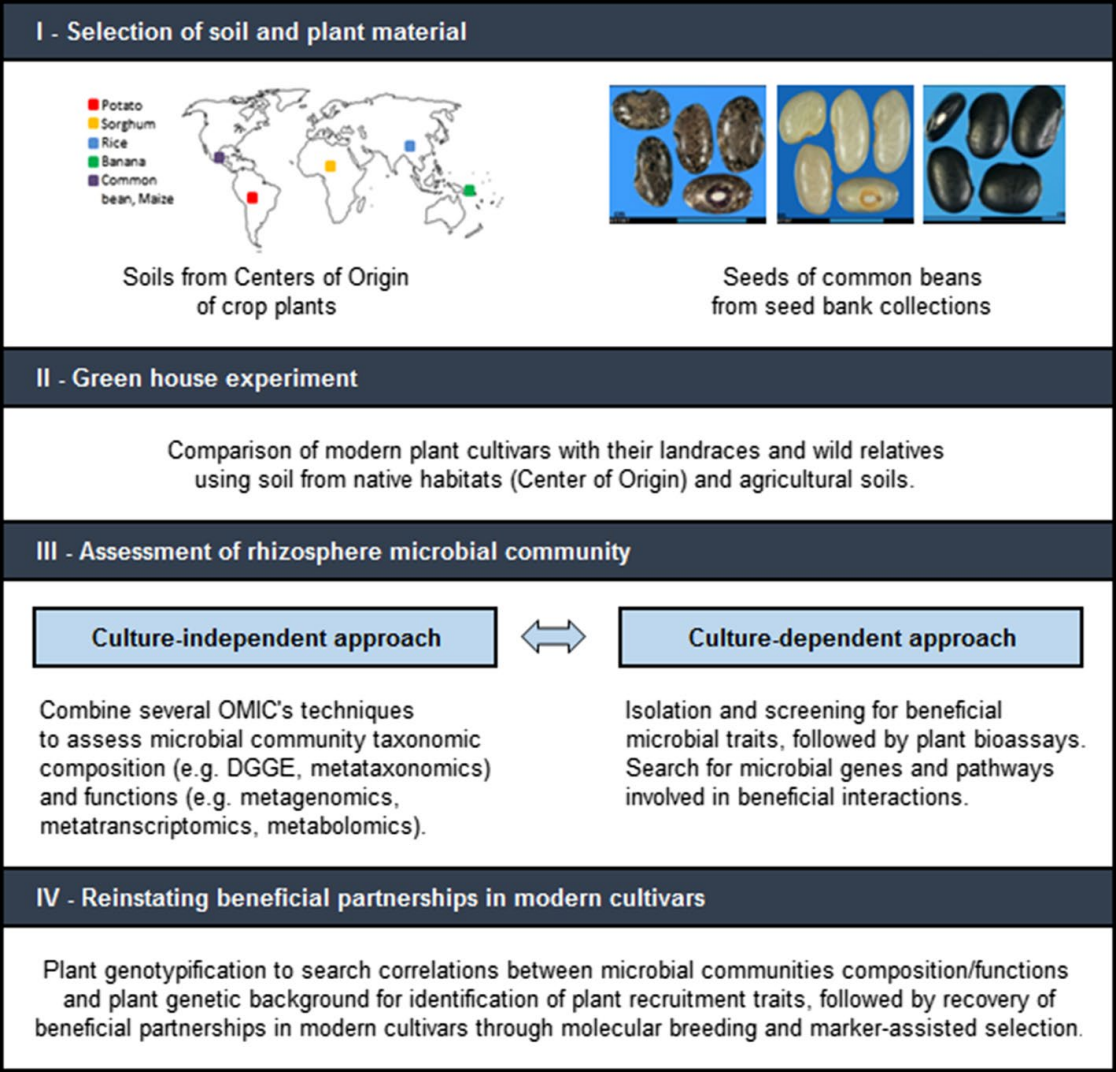
General workflow to investigate the possibility to reinstating beneficial partnerships in modern cultivars by assessing the rhizosphere microbiome of wild plants in native soil

Over the past decades, plant breeders have exploited genes from native relatives of modern crop species to improve plant growth and health. For instance, wild relatives have been used as sources of alleles to improve the ability of modern cultivars to withstand biotic and abiotic stresses in wheat (Nevo and Chen 2010; Budak et al. 2013; Placido et al. 2013), barley (Schmalenbach et al. 2008) and lettuce (Johnson et al. 2000; Simko et al. 2013). Similarly, entomologists have explored native habitats to identify natural enemies of insect pests. In the area of plant microbiome research, relatively few efforts have been made to study the biodiversity and functions of beneficial microbial communities present in the native habitats of ancestors of modern crop species. In a study comparing the microbiome of sugar beet and its ancestor *Beta vulgaris* spp. *maritime*, plants were grown in agricultural and in native soils (natural habitat). Wild beet plants showed a more diverse bacterial community compared with domesticated sugar beet, as was shown by single strand conformation polymorphism (SSCP) analysis of the 16S-rRNA genes from total community DNA and 16S amplicon pyrosequencing (Zachow et al. 2014). A first approach to identify plant loci associated with root colonization and pathogen protection by beneficial microorganisms involved a study with six inbred tomato lines and the biocontrol bacterium *Bacillus cereus* UW85 (Smith et al. 1997). Based on a dose–response model, they found differences for both intrinsic plant resistance to pathogen infection and support of biological control in the tomato lines. In a follow-up study with several recombinant inbred line (RIL) populations derived from an interspecific cross of cultivated tomato and the related wild species *Lycopersicon cheesmanii*, Smith and Goodman (1999) showed that several quantitative trait loci (QTL) were associated with support of growth of the biocontrol agent *B. cereus* UW85 and the disease suppressive effects. However, several conceptual and experimental efforts have yet to be made in order to identify and exploit these traits in a rhizosphere-based breeding program (Bakker et al. 2012). The search for plant traits linked to microbial recruitment by wild relatives holds a huge potential to elucidate and exploit beneficial interactions between plants and microbes. This hypothesis relies on the assumption that wild plant relatives have coevolved with the microbial community of native soils, performing an active selection of microbes with beneficial effects on plant growth and health. We hypothesize that wild relatives are able to establish, with higher frequency, beneficial interactions with microbes as compared to domesticated cultivars. In this context, we propose a pipeline for this emerging research area (Fig. 3). First, it is necessary to know the evolutionary history and process of domestication of the host plant to make a proper selection of wild plant materials, as well landraces and modern cultivars. If possible, the modern cultivars should be derived from the selected landraces; however this is not possible for all cultivated species, where the full domestication trajectory is unknown. In parallel, the centre of origin and centres of diversification should be known. Botanic and archaeological records have been used to determine where the wild relatives of many modern crops were originally formed as a species, followed by the domestication process and possible routes of dispersion by humans. Using this information, the collection of native soils in pristine sites located in the centre of origin and its use in the experimental setup will provide the native microbial assemblage in which wild relatives presumably recruit and sustain a more beneficial microbiome compared with less competent landraces and modern cultivars. To evaluate the impact of the different plant genotypes on the rhizosphere microbiome composition and functional potential, the use of metagenomics and metatranscriptomics together with culture-dependent approaches can be used to identify shifts in taxonomic and functional diversity of the microbiomes of the different plant genotypes. Based on these “omics” data, a screening with culture-dependent approaches can be performed by targeted isolation of those microbial genera that are specifically or more predominantly recruited by wild plant relatives. Evaluation of antagonistic activities against soil-borne pathogens, nutrient solubilisation or improved drought tolerance of the plant species after introduction will help to pinpoint those beneficial activities that plants look for in microbial partners. Finally, once the recruitment of particular taxa is confirmed and the utility of this association is determined, a plant genotyping strategy, QTL mapping and genome wide association studies (GWAS) with wild relatives, landraces, modern cultivars and preferably crosses between these plant genotypes must be performed in order to identify specific regions in the genome where the recruitment traits are located. Consequently, molecular breeding and marker-assisted selection can be applied to improve beneficial plant–microbe interactions in crop systems. Therefore, this approach of ‘going back to the roots’, i.e. assessing and accessing the microbiome of indigenous plants and their native habitats, represents a yet untapped avenue to further exploit microbes and plant traits in modern agriculture.
